# Research on Fiber-Optic Optical Coherence Ranging System Based on Laser Frequency Scanning Interferometry

**DOI:** 10.3390/s24061838

**Published:** 2024-03-13

**Authors:** Yingjian Zhou, Yanhong Yuan, Meixue Su

**Affiliations:** 1 School of Mechanical Engineering, Zhejiang Sci-Tech University, Hangzhou 310018, China; yingj_zhou@foxmail.com; 2 Zhejiang Light Industrial Products Inspection and Research Institute, Hangzhou 310018, China; julydeletter718@163.com

**Keywords:** frequency-scanning interferometry, distance measurement, Fourier transform, spectrum calibration

## Abstract

In this paper, a system for absolute distance measurement is proposed based on laser frequency scanning interferometry (FSI). The system utilizes a digitally tunable laser as the light source and employs synchronized pulses to drive an analog-to-digital converter (ADC) for interference signal acquisition. The frequency domain demodulation for absolute distance measurement is achieved through a three-spectrum line interpolation method based on the Hanning window. The system takes advantage of the spatial filtering characteristics of a single-mode optical fiber and the diffuse reflection properties of light to achieve a high integration of the prism system that forms the interference optical path. The resulting integrated fiber-optic probe is capable of measuring the distance to a non-cooperative target even when oriented at a certain angle with the target. We designed and fabricated a portable prototype. Experimental validation demonstrated that the maximum measurement distance of the system is 73.51 mm with a standard deviation of less than 0.19 μm for optimal measurement results. Even when there is an offset angle, the system maintains good measurement repeatability.

## 1. Introduction

The emergence of optical fibers in the 1970s propelled the development of optical measurement technology. Applications that utilize photons as carriers and optical fibers as mediums have expanded beyond information transmission into fields such as pressure, temperature, electromagnetic fields, and distance sensing [1,2,3,4]. Currently, absolute distance ranging methods based on optical technology include pulse-based methods, phase-based methods, femtosecond optical frequency comb methods, and laser frequency-scanning interferometry (FSI) [5,6,7,8]. Pulse-based methods are constrained by time accuracy, resulting in millimeter-level ranging accuracy. Phase-based methods are affected by the 2π phase ambiguity and often require multiple single-wavelength lasers to obtain integer orders. Benefiting from advancements in laser technology, the advantages of high precision and high resolution in distance measurement offered by femtosecond optical frequency comb methods and FSI are becoming increasingly apparent. Femtosecond optical frequency comb methods utilize femtosecond lasers based on mode-locking technology, using the optical frequency comb generated by these lasers as a light source for precise distance measurement. However, factors such as the high cost of optical frequency combs and the high environmental requirements for measurements limit the use of this technology in cost-sensitive or challenging environmental conditions. FSI modulates the frequency or wavelength of lasers continuously, utilizing the change in the interference phase caused by demodulating frequency sweeps to achieve absolute distance measurement. Compared to femtosecond optical frequency comb methods, FSI devices are simpler and more cost-effective, playing important roles in scientific research and engineering applications such as alignment and measurement in high-energy particle detectors like the Large Hadron Collider [9,10], general coordinate measurements [11,12], and optical coherence tomography scans [13,14].

**Figure 1:**
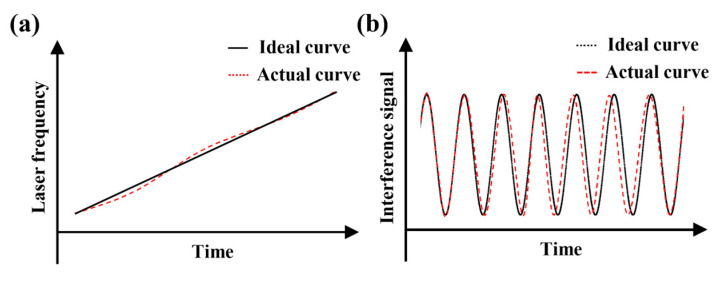
Waveforms of the frequency-scanning interferometry (FSI) ranging system. (**a**) Laser frequency-scanning waveform. (**b**) Interference light signal waveform.

Due to the inability of actual lasers to maintain a constant scanning speed during frequency scanning, laser frequency variation exhibits nonlinearity, as shown by the red line in Figure 1. Various scholars from different countries have conducted extensive research on how to reduce the errors introduced by frequency scanning nonlinearity to improve the measurement accuracy of FSI. Currently, correction techniques for the nonlinear frequency scanning of lasers can be roughly divided into two categories. One is an active frequency stabilization technology that maintains the laser output frequency at a stable reference value through a feedback mechanism. In 2009, Roos et al. [15] employed a fast–slow dual-loop optoelectronic phase-locked loop to simultaneously correct the current and piezoelectric ceramics of an external cavity frequency-scanning semiconductor laser. Within a range of 1.5 m, the distance resolution reached up to 31 μm. In 2015, Kakuma et al. [16] stabilized the sweep speed of the light source using a circuit controller based on a phase-locked loop technology. They marked the frequency using a rubidium gas cell (Rb-cell) and achieved a sub-micrometer measurement accuracy within a 14 mm measurement range, with a measurement standard deviation of 0.12 μm. In 2021, Deng et al. [17] used the linear drive signal and instantaneous optical frequency of an external cavity laser diode as the input and output data of an asymmetric Prandtl–Ishlinskii hysteresis model. They utilized the inverse function of this model as a feedforward controller to suppress the nonlinearity of frequency scanning. The root mean square error in the simulation results was 1.637 × 10^−6^ GHz. Another category involves tracking the frequency of interference signals and using algorithms to extract and correct nonlinearity through passive compensation techniques. In 2009, Yüksel K et al. [18] utilized peak and valley points with intermediate frequency spacing in auxiliary interference signals to resample the measured interference signals. The resolution of the half-width at the half-maximum of the resampled frequency domain peaks was 0.4 cm, and the spatial resolution was 30 times that before resampling. In 2014, Shi et al. [19] made improvements to the auxiliary interference optical path, achieving a measurement resolution of 50 μm within a 10 m measurement range. In 2019, Jiang et al. [20] employed a method based on Hilbert phase unwrapping to subdivide equal-frequency intervals, effectively reducing the need for the delayed fiber length in the auxiliary optical path. Within a 5 m measurement range, the standard deviation of the measurement results was 4.64 μm. In 2022, Wang et al. [21] efficiently eliminated the nonlinearity of a frequency-modulated fiber laser using equifrequency resampling techniques. They achieved a measurement standard deviation of less than 0.07 mm at an output power below 1 mW for distances up to 152 m.

It can be observed that the current ranging systems using FSI still face challenges such as bulky interference optical paths composed of lens systems, inconvenience in portability, the need for improvement in measurement accuracy, and high costs. In this study, based on the principle of FSI, a digitally tunable laser is employed as the light source. The interference signal is collected by synchronously driving an analog-to-digital converter (ADC) with pulsed signals from the laser tuner. The Fourier transform and spectral correction methods are utilized to demodulate the collected interference signals. Simultaneously, the lens system is highly integrated to provide a device for absolute distance measurement that is applicable for non-cooperative target detection, high precision, and multi-channel measurements. The designed and fabricated portable prototype features a compact probe volume and multi-channel capability, making it suitable for measuring object contours in scenarios inaccessible to conventional cameras. Moreover, the system imposes no strict requirements on the angle between the fiber probe and the surface of the object being measured. This allows the system to utilize sensitive elements to convert physical quantities into deformations and then indirectly measure physical quantities such as pressure, temperature, and strain by measuring the deformations using this system.

## 2. Materials and Methods

### 2.1. The Basic Principle of FSI

The basic optical path of the FSI measurement system is derived from the Michelson interferometer proposed by American physicists Michelson and Morley in 1883. Assuming the intensity of the reference light is $I_{1}$, the intensity of the measurement light is $I_{2}$, and the distance to be measured is L, the intensity of the interference light I formed can be expressed as:

(1) $$I=I_{1}+I_{2}+2\sqrt{I_{1}I_{2}}\cos(\frac{4\pi Lv}{c}+\phi )$$

where v is the instantaneous laser frequency, c is the speed of light, and ϕ is the initial phase of the interference light. As per Equation (1), it can be observed that when the laser frequency changes linearly, the obtained interference light intensity also exhibits a periodic signal, as shown in Figure 1. Assuming a frequency scanning period, the change in the laser frequency Δv and the change in the interference signal phase Δθ are given by

(2) $$L=\frac{c\Delta \theta }{4\pi \Delta v}$$

In the case where the change in the laser frequency is known, calculating the distance to be measured, denoted as L, only requires obtaining the change in the phase of the interference signal, denoted as Δθ. Therefore, the measurement accuracy of distance L depends on the measurement accuracy of the phase change Δθ.

### 2.2. System Description

#### 2.2.1. System Composition

**Figure 2:**
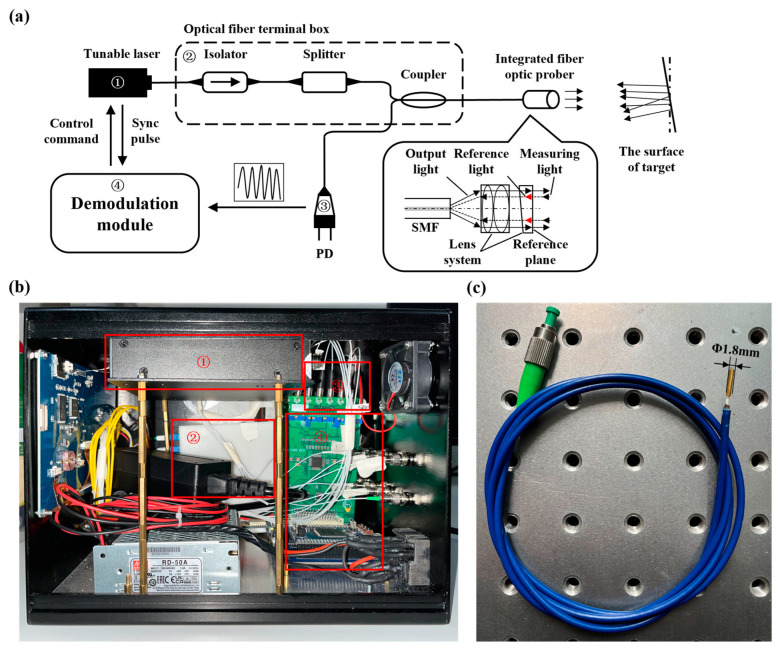
Research into fiber-optic optical coherence ranging system based on FSI. (**a**) Schematic diagram. (**b**) Photo of the demodulator. (**c**) Photo of the integrated fiber probe.

The experimental prototype of the fiber-optic optical coherence ranging system based on FSI is shown in Figure 2. For ease of identification, the device numbers in the schematic and the physical diagram are the same. The system consists of a digitally tunable laser (CA92004, UC INSTRUMENTS, Fremont, CA USA), fiber isolator (Jiayitong Technology Co., Ltd., Shenzhen, China), fiber splitter (Jiayitong Technology Co., Ltd., Shenzhen, China), fiber coupler (Jiayitong Technology Co., Ltd., Shenzhen, China), integrated fiber probe, photodiode (HAMAMATSU, Beijing, China), and demodulation module. The demodulation module controls the digitally tunable laser to emit a laser with a tunable frequency. The laser is transmitted through the fiber isolator, fiber splitter, and fiber coupler to reach the integrated fiber probe. In the integrated fiber probe, part of the laser is reflected by a prism as the reference light, while the other part is reflected by the surface of the measured object as the measurement light. The interference light is formed when these two parts of the light meet. The interference light is transmitted through the fiber coupler to reach the photodiode, where the photodiode converts the interference light signal into an electrical signal. The demodulation module processes and amplifies the interference signal and collects the interference voltage signal based on the synchronous pulse of the digitally tunable laser. At the end of a frequency scanning cycle, the demodulated interference signal can extract the phase difference information between the reference light and the signal light, thus achieving absolute distance measurement. The fiber isolator, fiber splitter, and fiber coupler are encapsulated in the fiber terminal box. The role of the fiber isolator is to prevent the light transmitted back from the fiber coupler from entering the laser, thereby avoiding optical crosstalk inside the laser. The experimental prototype utilizes a fiber splitter to divide the light emitted by the laser into 8 channels, thereby achieving the synchronous measurement of distance values for 8 channels.

#### 2.2.2. Integrated Optical Fiber Probe

Traditional coherent optical ranging methods require the measured surface to be smooth, forming a planar reflection. Strict alignment of the optical path is essential, and even slight angular deviations can lead to measurement failures. Therefore, when the measured surface cannot reflect light like an optical surface, it is common to install a reflector on the measured surface. Therefore, when the surface being measured cannot reflect light like an optical surface, people typically need to install a reflector on the surface in order to achieve distance measurement by collaborating with the reflector. The integrated fiber probe of this system utilizes the spatial filtering properties of single-mode optical fibers and the diffuse reflection characteristics of light. This design allows for less stringent alignment of the optical path during application, enabling measurements on non-optical surfaces.

When the measured surface resembles the surface of the metal being processed with a certain roughness, the light incident on its surface at any angle will generate scattered light within a large spatial angle range. The direction of propagation of the scattered light is dispersed, resulting in low coherence. It is difficult for the reference light and the signal light scattered by the measured surface to interfere with each other and form interference fringes, making interference measurement impossible. The core of a single-mode optical fiber is only about 9 μm in diameter, making it an excellent spatial filter. If the reference light and the signal light scattered by the measured surface are simultaneously coupled into the core of the optical fiber, the spatial coherence of the signal light is restored by the optical fiber core’s spatial filtering. The two parts of light will interfere well, allowing the interference light reaching the photodiode to carry precise information about the position of the scattering points.

As shown in Figure 2a, the integrated fiber probe integrates the lens system of the interference optical path, consisting of a single-mode optical fiber (Zhonghui Photoelectric Technology Co., Ltd., Dongguan, China), two spherical lenses (Zhonghui Photoelectric Technology Co., Ltd., Dongguan, China), and a prism with a small angle (Zhonghui Photoelectric Technology Co., Ltd., Dongguan, China). In addition to collimating the light diverged from the core of the optical fiber, the spherical lenses focus and couple the light reflected from the measured target surface back into the optical fiber. All surfaces of the spherical lenses are coated with anti-reflective films, so the power of the light reflected from each surface and coupled back into the core of the optical fiber can be ignored. The angle of the prism with a small angle is 8 degrees. When collimated light enters the prism, the incident surface is at a certain angle to the optical axis and coated with an anti-reflective film. The light reflected from its surface cannot be recoupled into the core of the optical fiber. The exit surface of the prism serves as the reference surface, reflecting some of the light back into the core of the optical fiber as reference light, with a reflection ratio of about 4%. The diameter of the integrated fiber probe is only 1.8 mm, with a length of 10 mm, making it easy to install in various measurement situations. Even if there is a certain angular offset between the probe and the measured surface, it can still complete non-cooperative distance measurements.

#### 2.2.3. Interference Signal Acquisition

This system utilizes a digitally tunable laser as its light source. In the scanning mode, this laser can output laser beams with a frequency (wavelength) that jumps at regular intervals. The maximum range of the laser frequency output is from 191,200 to 196,500 GHz, with a minimum step size of 1 GHz. The wavelength output has a maximum range of from 1525 to 1568 nm. The laser is equipped with an internal constant temperature control circuit, ensuring wavelength stability and repeatability at ±2 pm. The maximum output power of the laser is 20 mW, and a single laser can serve as the light source for dozens of interference optical paths through spectral splitting.

The ranging system’s demodulation module consists of a data acquisition card based on an 8-channel, 16-bit ADC chip (MAX11046, Maxim Integrated, San Jose, CA, USA) with a range of from 0 to 5 V and a main control board based on an STM32F407 chip (STM32F407ZGT6, STMicroelectronics, Plan-les-Ouates, Switzerland). The data acquisition card performs computation, amplification, and ADC on the interference electrical signals converted from the photodiode. The main control board oversees the overall control of the system and demodulation of the interference signals. The laser provides feedback on the current frequency modulation operation. During the frequency scanning process, with each step forward, the Trig port of the laser outputs a synchronized pulse signal. The interval delay (Sync delay) and the holding time (Dwell) of the synchronized pulse can be configured. Therefore, the period of the synchronized pulse emitted by the laser is the sum of the laser completing one step frequency sweep, the interval delay, and the holding time. The frequency-scanning laser output by this laser will have better linearity and center frequency repeatability. By driving the ADC based on synchronization pulses to collect interference signals, it is advantageous for improving the measurement accuracy and repeatability of the system.

**Figure 3:**
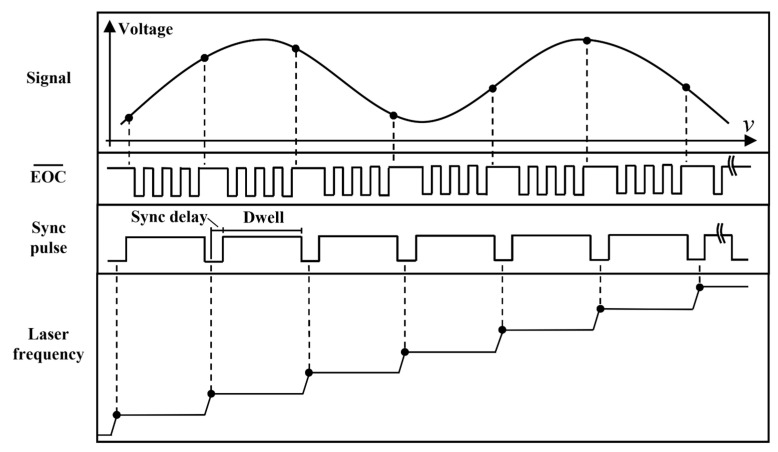
Schematic diagram of system sampling timing.

As shown in Figure 3, after completing one step of frequency variation, the laser waits for a Sync delay time and then generates a high-level signal with a duration of Dwell. Based on this signal, the ADC drives the collection and conversion of interference signals. Once the ADC completes the conversion, it pulls down the EOC pin. During the high-level signal holding time, the interference signal is continuously collected and converted four times, and the average value is taken as the intensity of the interference signal corresponding to that laser frequency. Therefore, at the end of one frequency scanning cycle of the laser, the system collects data points with the laser frequency as the x-axis and the voltage value (Voltage) as the y-axis, where the voltage value corresponds one-to-one with the known and determined laser frequency.

The interference optical signal is converted into an analog current signal after reaching the photodiode. Note that the photodetector chosen has a photoresponsivity of 1.25 A/W. Due to the splitting of the laser emitted by the laser and the scattering losses on the surface of the measured target through the fiber splitter, the actual interference light intensity reaching the photodiode is only at the μW level or even lower. Consequently, the current resulting from the photoelectric conversion is also in the μA range or even lower. Such a weak electrical signal is challenging to collect. Therefore, it is necessary to utilize operational amplifier circuits (AD8667, Analog Devices, Inc., Wilmington, MA, USA) for signal processing, converting and amplifying the current signal into a voltage signal at the mV level or higher. Let the current output by the photodiode be denoted as $I_{0}$, and let the voltage output after amplification by the operational amplifier circuit be denoted as $U_{0}$. Then, there exists the following relationship between $I_{0}$ and $U_{0}$:

(3) $$U_{0}=I_{0}R$$

In the equation, R represents the amplification resistor. To fully utilize the range of the ADC, ensuring the accuracy of the collected signal without causing overflow during ADC acquisition, this system selects a resistor with a resistance of 10 MΩ (Uniroyal Electronics Global Co., Ltd., Kunshan, China) and a variable resistor with a maximum resistance of 1 MΩ (Uniroyal Electronics Global Co., Ltd., Kunshan, China) in series as the amplifying resistors for the operational amplifier circuit.

### 2.3. Signal Demodulation Methods

The digital tunable laser is set with a scanning start frequency of 191,500 GHz, an end frequency of 195,700 GHz, and a scanning step of 1 GHz. Therefore, at the end of one frequency scanning cycle, the system will collect 4200 data points. The Fourier transform is applied to demodulate the interference signal. To reduce computational complexity and enhance efficiency, a power of two was chosen for the Fourier transform, specifically considering signals corresponding to points from 1 to 4096 as useful. As processors can only handle discrete and finite-length data, a discrete form of the Fourier transform is employed here:

(4) $$I\left(m,L\right)=I_{1}+I_{2}+2\sqrt{I_{1}I_{2}}\cos(\frac{4\pi L}{c}(v_{0}+mv_{s})+\phi )\;\;\;\;\;\;\;m=0,1,\ldots N-1$$

In the equation, $v_{0}$ represents the initial laser frequency, $v_{s}$ is the laser frequency scanning interval, and N is the number of sampling points. The interference signal, after removing the DC component, is represented in a discrete form:

(5) $$I\left(m,L\right)=2\sqrt{I_{1}I_{2}}\cos(\frac{4\pi L}{c}(v_{0}+mv_{s})+\phi )\;\;\;\;\;\;\;m=0,1,\ldots N-1$$

The discrete Fourier transform (DFT) is expressed as:

(6) $$X(k)=\sum_{m=0}^{N-1}I(m,L)exp(-j\frac{2\pi }{N}mk)\;\;\;\;\;\;\;k=0,1,\ldots N-1$$

After the discrete Fourier transform, the frequency index $k_{d}$ corresponding to the maximum value X(k) can be identified, and the measured distance value can be expressed as:

(7) $$L=\frac{ck_{d}}{2Nv_{s}}$$

From Equation (7), it is evident that when the number of sampled points is constant, the resolution of the distance measurement system depends on the resolution of $k_{d}$. The quantization effect of the frequency spectrum after discretization results in values of $k_{d}$ being restricted to integers. To achieve higher resolution, it is necessary to interpolate the spectrum after the discrete Fourier transform. In this paper, a three-spectrum line interpolation method based on the Hanning window is employed to correct the spectrum of the interference signal [22]. The formula for calculating the normalized frequency correction term is:

(8) $$\gamma =\frac{1.5A_{am1}(A_{am11}-A_{am12})}{\left(A_{am1}+A_{am11})(A_{am1}+A_{am12}\right)}$$

In the formula, $A_{am1}$, $A_{am11}$, and $A_{am12}$ represent the amplitudes of the maximum amplitude spectrum lines $k_{d}$, $k_{d}+1$, and $k_{d}-1$, respectively. The corrected value of the distance to be measured is:

(9) $$L=\frac{c(k_{d}+\gamma )}{2N\Delta v}$$

**Figure 4:**
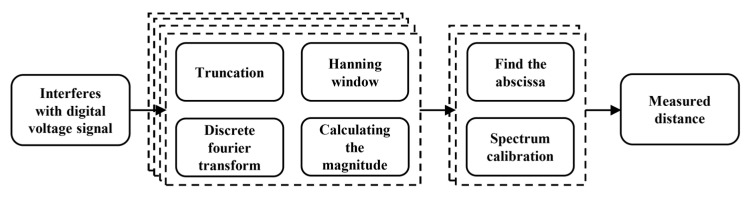
Schematic diagram of the interference signal demodulation process.

The demodulation process of the collected interference signal in this system is illustrated in Figure 4.

According to the Nyquist sampling theorem, to accurately recover the original continuous signal from the sampled signal, the sampling frequency must be at least twice the highest frequency component of the signal. According to Equation (4), the minimum step size for laser frequency scanning is 1 GHz. Calculating with the sampling frequency being twice the frequency of the interference signal, theoretically, this system can achieve a maximum absolute distance measurement of 75 mm.

## 3. Results

**Figure 5:**
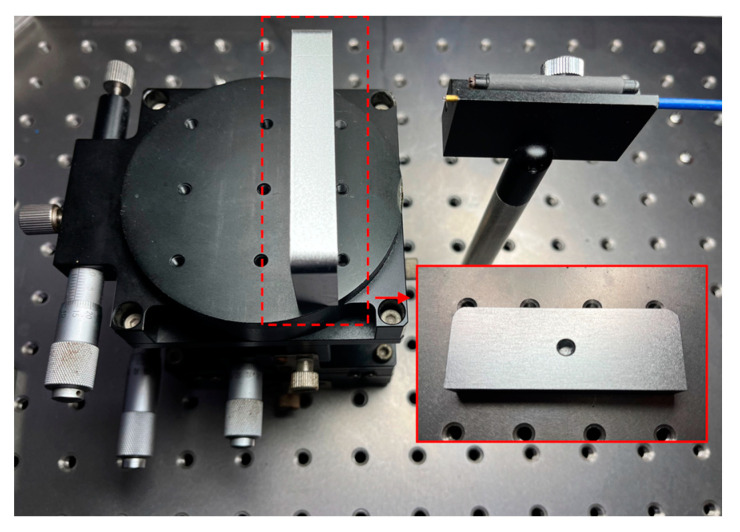
The measured component.

**Figure 6:**
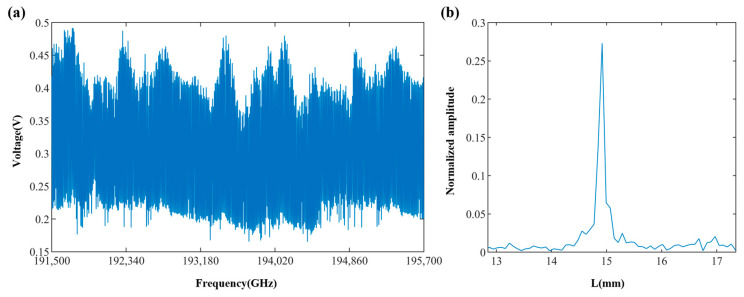
Complete interference signal over one scanning cycle. (**a**) Interference signal waveform. (**b**) Signal spectrum.

The experimental setup of this system is illustrated in Figure 2a, where the completed experimental prototype comprised a demodulator and an integrated fiber probe, as depicted in Figure 2b,c. The integrated fiber probe was mounted on an optical experimental platform (Hengyang Electronic Technology Co., Ltd., Shenzhen, China) to mitigate the effects of vibration, and using this setup, we measured the distance from the integrated fiber probe to the surface of the target object. The target object (see Figure 5), made of aluminum alloy, exhibited machining marks and had not undergone polishing treatment, with its surface roughness ranging from Ra = 12.5 μm to Ra = 6.3 μm, indicating it was not optically smooth. The system took approximately 1 s to complete one distance measurement. Figure 6a shows the interference signal waveform acquired by the measurement system over one frequency scanning cycle at a distance of approximately 14.85 mm with an offset angle of 0 degrees. From the waveform, it is evident that under the conditions of the measured surface being non-optical, the reference light reflected by the prism and the signal light scattered by the measured surface could form a good interference pattern. By demodulating the collected interference signal, the distance value of this measurement could be calculated. Figure 6b presents the spectrum of the interference signal at this distance.

**Figure 7:**
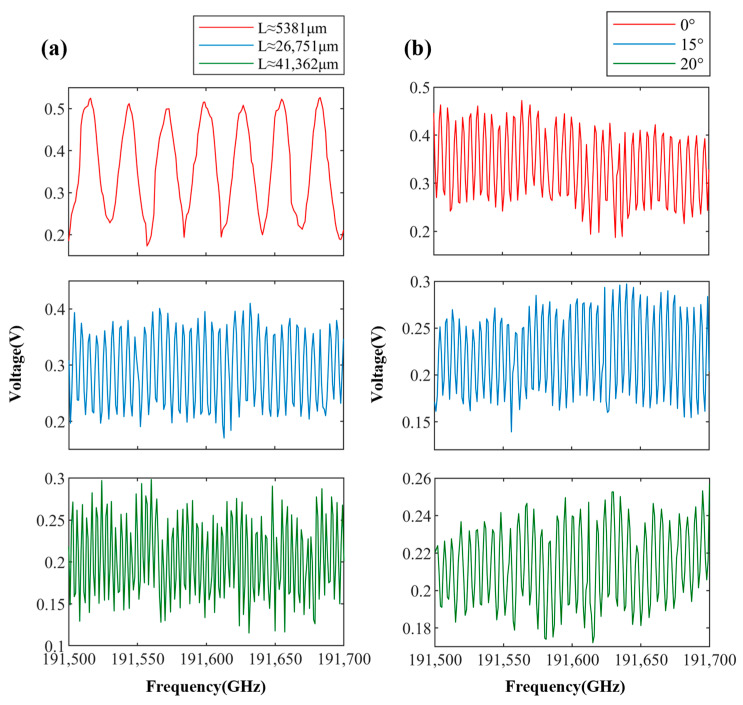
Interference signal waveforms. (**a**) Interference signals at different distances with an offset angle of 0 degrees. (**b**) Interference signals at a distance of 22 mm with different offset angles.

Absolute distance measurements on the surface of the metal were conducted at different distances and angles. For ease of observation, the first 200 points of the interference signal were selected, resulting in the waveform plots shown in Figure 7. Figure 7a illustrates the interference signal waveforms at different distances with an offset angle of 0 degrees, while Figure 7b shows the interference waveforms at a distance of 22 mm with varying offset angles. It can be observed that the voltage amplitude of the interference signal was inversely proportional to the measurement distance and offset angle. An increase in the measurement distance or offset angle led to a decrease in the voltage amplitude of the interference signal. However, even in the presence of certain offset angles, the acquired waveforms could still be demodulated for absolute distance determination.

**Figure 8:**
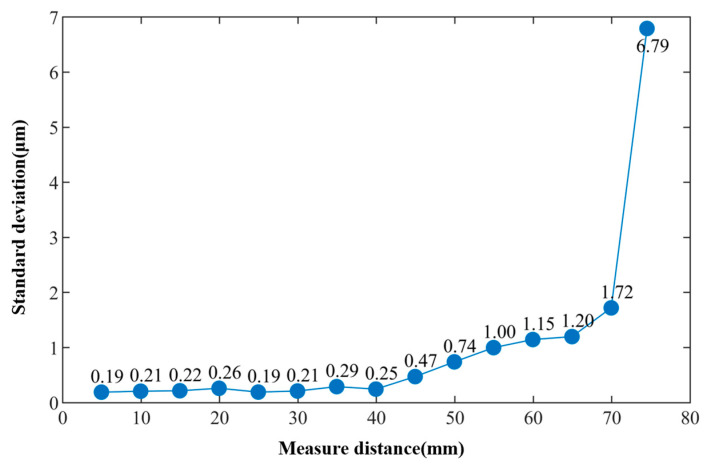
The standard deviations of measurement results at different distances with an offset angle of 0 degrees.

The target object was fixed on a displacement platform, maintaining an offset angle of 0 degrees, to conduct experimental tests on the measurement repeatability of the system at different distances. Initially, the distance between the integrated fiber probe and the target surface was 4.91 mm. Subsequently, the displacement platform (Runjia Pneumatic Technology Co., Ltd., Yueqing, China) was moved continuously in 5 mm increments, and distance measurements were taken continuously 100 times after each movement. After the 14th movement of the displacement platform, the distance exceeded the measurement range of the system. By manually adjusting the displacement platform, the maximum distance that the system could measure under this condition was determined to be 73.51 mm. Repeat measurements were also conducted at this distance, resulting in a total of 15 sets of measurement data. The repeatability standard deviations corresponding to the different distances obtained from the experiment are shown in Figure 8. It can be observed that within a distance of 40 mm, the standard deviations of the measurement results at each distance were relatively small, all remaining within 0.29 μm, with the smallest standard deviation reaching 0.19 μm. However, beyond 40 mm, the repeatability standard deviation increased with the increase in distance.

**Figure 9:**
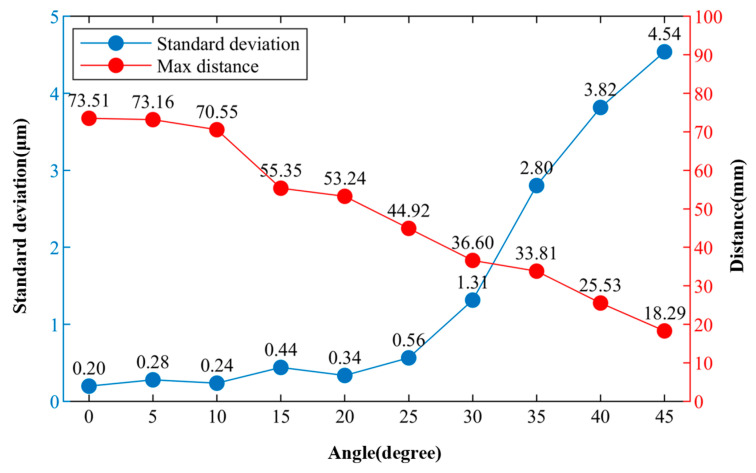
The standard deviations of measurement results and the corresponding maximum measurable distances at different offset angles when the distance was 10 mm.

The target object was similarly fixed on a displacement platform to conduct experimental tests on the measurement repeatability of the system and the maximum measurable distance at different offset angles. The experimental range of offset angles was from 0 degrees to 45 degrees, with increments of 5 degrees. During the repeatability test, the distance was maintained at around 10 mm, and measurements were taken continuously 100 times for each angle. Figure 9 shows the standard deviations of measurement results and the corresponding maximum measurable distance at different offset angles when the distance was 10 mm. It can be observed that even with certain offset angles, the system maintained good repeatability. As the offset angle increased, the stability of the system and the maximum measurable distance showed a decreasing trend.

## 4. Discussion

The digital tunable laser used in this system has a high output power and precise frequency positioning. However, due to its minimum scanning step size of 1 GHz, the measurement range of the system is limited. The experimental results show that the maximum measurable distance of the system is 73.51 mm, which is close to the theoretical value calculated based on a sampling frequency that is twice the frequency of the interference signal. However, as the sampling ratio decreases, the signal acquired by the system may experience quality degradation or even information loss. This is the reason for the significant increase in the standard deviation of the measurement results at a distance of 73.51 mm. Choosing a laser with a smaller scanning step size can increase the measurement range of the system.

The maximum offset angle for distance measurement depends on the surface of the object being measured. As long as the light scattered from the surface and coupled back into the fiber has sufficient intensity compared to the reference light, the system can perform distance measurement at that offset angle. However, as the offset angle increases, the intensity of the scattered light coupled back into the fiber decreases, resulting in a reduction in the intensity of the interference light. When the intensity of the interference light is too low, the interference signal will be submerged in noise, making it difficult to accurately extract distance information from the background noise. This is also the reason why, in the experiment, as the offset angle increased, the stability of the system decreased and the maximum measurable distance decreased.

However, in practical measurements, environmental light, temperature drift in the acquisition circuit, and vibrations can impact the collected interference signal. The tiny vibration displacement of the measured object can be amplified by tens or even hundreds of times, which is a major source of measurement error during experiments [23,24]. Shortening the measurement cycle of the system and compensating for vibration errors during the frequency scanning process will be a key focus for improving the measurement accuracy of this system in the future.

## 5. Conclusions

In this paper, we propose an absolute distance measurement system based on FSI. It utilizes a digitally tunable laser as the light source and drives the ADC to collect interference signals through synchronous pulses, effectively reducing measurement errors caused by non-linearities in frequency scanning. To address the drawbacks of the interference optical path’s large volume due to the lens system, we achieved a high level of integration of the prism system using the spatial filtering characteristics of a single-mode optical fiber and the diffuse reflection properties of light. The resulting integrated fiber-optic probe is capable of measuring the distance to a non-cooperative target even when oriented at a certain angle with the target. By using an optical fiber splitter to divide the laser emitted by the laser into multiple channels, the system can simultaneously measure distances in up to eight channels, reducing the cost of laser usage. We designed and fabricated a portable prototype. The experimental validation demonstrated that the maximum measurement distance of the system is 73.51 mm, with a standard deviation of less than 0.19 μm for optimal measurement results. Even when there is an offset angle, the system maintains good measurement repeatability.

## Data Availability

Data are contained within the article.
